# The Mission Parcel Society

**Published:** 1887-09-03

**Authors:** Mary E. Webster

**Affiliations:** Ripple Lodge, Malvern Link


### THE MISSION PARCEL SOCIETY.

Sir,—Will you kindly give me space for a few words asking those of your readers who are willing to do something for our missionaries to post regularly one or more of their newspapers and magazines, when read, to an address which I will gladly supply, together with postal rates, etc., on receiving from them a stamped envelope and the name of the paper offered?

I keep a register containing the addresses of missionaries and a list of the papers each receives, also what each specially asks for. Letters are constantly arriving from abroad full of gratitude and appreciation for this little act of kindness, and are quite sufficient to repay senders for the small trouble or expense they incur.

Mary E. Webster.

Ripple Lodge, Malvern Link.

